# Desmopressin in the treatment of nocturnal enuresis in patients with spina bifida

**DOI:** 10.1186/1743-8454-7-S1-S10

**Published:** 2010-12-15

**Authors:** Pieter Dik, Paul W Veenboer, Tom PVM de Jong

**Affiliations:** 1 Wilhelmina Children’s Hospital, Lundlaan 6, Â 3584 EA Utrecht, PO BOX 85090, The Netherlands

## Background

It was observed that some of our patients with spina bifida (SB) that became dry during daytime still suffered urine loss during the night. It was difficult to wake them up. It seemed that they suffered from normal nocturnal enuresis (NE).

## Materials and methods

Of 241 SB patients that are in yearly follow-up in our institution, 203 patients were 5 years or older and 13 of them seemed to have true NE. These 13 patients were all treated with Desmopressin 0,4 mg ante noctem.

## Results

Thirteen patients, 5 males and 8 females were treated with Desmopressin and they were evaluated. Nine patients were operated beforehand: 8 patients were treated with a bladder augmentation and bladder neck sling suspension and one boy had an urethral valve resection. All 13 patients were dry during daytime and 3 of them even did not need antimuscarinic agents. All 13 patients had normal kidney function and urodynamic studies showed compliant bladders without overactive contractions. During nighttime however they wetted their beds. Desmopressin (0,4 mg a.n.) was successful in 12 patients: they became completely dry during the night. One of them however relapsed and he will be treated with an alarm clock system as an adjuvant therapy.

## Conclusions

Until now NE was not recognised as a possible cause of nocturnal urine loss in SB patients because the incontinence was considered to be caused by overactive neuropathic bladder behaviour. Desmopressin as a monotherapy was successful in 11/13 SB patients with recognised NE.

